# Physics-informed machine learning analysis for nanoscale grain mapping by synchrotron Laue microdiffraction

**DOI:** 10.1107/S160057672500826X

**Published:** 2025-10-18

**Authors:** Ka Hung Chan, Xinyue Huang, Nobumichi Tamura, Xian Chen

**Affiliations:** a Department of Mechanical and Aerospace Engineering, The Hong Kong University of Science and Technology, Hong Kong; b Advanced Light Source Lawrence Berkeley National Laboratory Berkeley USA; HPSTAR and Harbin Institute of Technology, People’s Republic of China

**Keywords:** synchrotron Laue microdiffraction, physics-informed machine learning, nanocrystals, grain mapping, X-ray crystallography

## Abstract

Understanding the grain morphology, orientation distribution and crystal structure of nanocrystals is essential for optimizing the mechanical and physical properties of functional materials. Synchrotron X-ray Laue microdiffraction is a powerful technique for characterizing crystal structures and orientation mapping using focused X-rays. However, when the grain sizes are smaller than the beam size, mixed peaks in the Laue pattern from neighboring grains limit the resolution of grain morphology mapping. We propose a physics-informed machine learning (PIML) approach that combines a convolutional neural network feature extractor with a physics-informed filtering algorithm to overcome the spatial resolution limits of X-rays, achieving nanoscale resolution for grain mapping. Our PIML method successfully resolves the grain size, orientation distribution and morphology of Au nanocrystals through synchrotron microdiffraction scans, showing good agreement with electron backscatter diffraction results. This PIML-assisted synchrotron microdiffraction analysis can be generalized to other diffraction-based probes, enabling the characterization of nanosized structures with micrometre-sized probes.

## Abstract 2

Our work presents a physics-informed machine learning approach that advances materials characterization by overcoming the fundamental resolution limits of synchrotron X-ray Laue microdiffraction. By integrating deep learning with physical constraints, we demonstrate that nanoscale grain mapping can be achieved even when using micrometre-sized X-ray probes.

## 1. Introduction

Morphological configurations of polycrystals, including phase interfaces, grain boundaries, orientation distribution and textures, play a profound role in the mechanical and physical properties of functional materials. The nanoscale grains and precipitates underlie revolutionary technological advancements such as solar cells (Wang *et al.*, 2023 ▸; Kodalle *et al.*, 2024 ▸), plasmonic devices (Zgrabik & Hu, 2015 ▸; Bi *et al.*, 2021 ▸) and thermoelectric devices (Liu *et al.*, 2018 ▸; Ojo *et al.*, 2021 ▸). A comprehensive analysis of the morphological features of grains and heterogeneous phases is necessary for the development of novel materials with tailored properties. Laue microdiffraction experiments are widely utilized to characterize crystallographic information, grain orientations, localized strains, heterogeneous phases and precipitates, but their spatial resolution is fundamentally limited by the size of the focused X-ray probe (Chen *et al.*, 2016 ▸).

Nanocrystalline materials with grain sizes below 500 nm are typically difficult to detect using a micrometre-sized polychromatic X-ray beam, even when it is generated by a synchrotron source. Recent advances have pushed the optical focusing limit of photon flux to achieve submicrometre beam sizes (*i.e.* 300 nm) (Liu *et al.*, 2011 ▸; Ulrich *et al.*, 2011 ▸), but resolving grains at the nanoscale remains challenging due to the Laue pattern consisting of peaks diffracted from multiple neighboring grains. While use of new and better X-ray optics might be able to solve this issue, this approach is not ideal for existing synchrotron facilities. Firstly, ultra-precise optical mirrors that produce 100 nm beams are costly and require substantial modifications to the beamline (Tucoulou *et al.*, 2008 ▸; Nazaretski *et al.*, 2017 ▸; Mino *et al.*, 2018 ▸). These mirrors are also more difficult to maintain and align, as their performance typically degrades over time. Not all facilities can afford these upgrades. Additionally, even with a very small beam, the problem of X-ray penetration remains.

Conventional X-ray crystallographic analysis relies on Laue indexing for a set of diffracted peaks from a single orientation (Tamura, 2014 ▸). However, this method is not sufficient to resolve grains with sizes smaller than the beam size, as one Laue pattern could contain many orientations. Even advanced algorithms like the synchrotron *X-ray Microdiffraction Analysis Software* (*XMAS*) developed by the Advanced Light Source (ALS) (Tamura, 2014 ▸) and *LaueTools* and *LaueNN* developed by the European Synchrotron Radiation Facility (Robach & Micha, 2015 ▸; Purushottam Raj Purohit *et al.*, 2022 ▸), which can distinguish the superposition of Laue indexing for multiple grains, have their limitations. For instance, *LaueNN*, a sophisticated neural-network-based algorithm, can refine the indexing for about ten grains, but this is still not enough to resolve nanosized grains. Moreover, such sequential indexing procedures are computationally intensive and time consuming.

To address the challenges of synchrotron Laue microdiffraction experiments and their analytical approaches for nanocrystals or nanocrystalline functional materials, a fundamental question arises: is it necessary to obtain Laue indexing for each pattern if the ultimate goal is to understand the material’s morphological configurations? Laue patterns diffracted by a synchrotron white beam with a sufficiently wide energy bandpass contain hidden features that can be directly utilized for spatial segmentation of grains with different orientations. According to our previous work on indexing-free analysis of synchrotron Laue diffraction (Song *et al.*, 2019 ▸), the Laue pattern can be encoded into 256 latent features by the convolutional neural network autoencoder *CANON*.^1^ Unlike conventional methods, *CANON* skips the Laue indexing procedure and directly generates the grain map from the learned latent features. By reducing each image to a feature vector in a lower-dimensional latent space, this method significantly accelerates data processing, especially when handling large datasets. While the *CANON* analysis package shows promise in extracting hidden features from Laue patterns and bypassing the indexing process, its ability to resolve grains smaller than the X-ray beam size remains uncertain. The potential of *CANON* to analyze Laue patterns with superpositions of many nanoscale grains without indexing and to identify the primary grain among numerous satellite grains exposed by micrometre-sized X-ray beams is intriguing. Yet, the superposition of multiple reflections results in complex, nonlinear combinations of features that the autoencoder struggles to disentangle. To address this challenge, incorporating additional physical information, such as peak intensity distribution, could enhance the machine learning (ML) algorithm’s ability to resolve features within Laue patterns. This approach may further increase the spatial resolution of micrometre-sized X-ray beams, enabling the determination of submicrometre to nanoscale grain morphological distributions.

In this paper, we present a physics-informed data filtering and pooling algorithm designed for feature engineering to effectively isolate diffraction peaks from primary orientations in Laue patterns. The proposed algorithm generates a set of refined patterns that concentrate on the peak intensities corresponding to the primary nanograin within the exposure domain, which is illuminated by focused white-beam syn­chro­tron X-rays. This method systematically removes stray peaks originating from less-exposed grains, thereby facilitating the feature learning process by *CANON*. The processed Laue patterns markedly enhance the orientation resolution of local nanograins, a critical factor for subsequent analysis using the physics-informed machine learning (PIML) framework. We validate our PIML approach through its application to a nanocrystalline gold thin film deposited on a single-crystal silicon substrate. The thin film has a thickness of 1 µm and features grain sizes on the nanometre scale.

## 2. Experiments and methods

### 2.1. Setup and philosophy of X-ray microdiffraction for grain mapping

**Figure 1:**
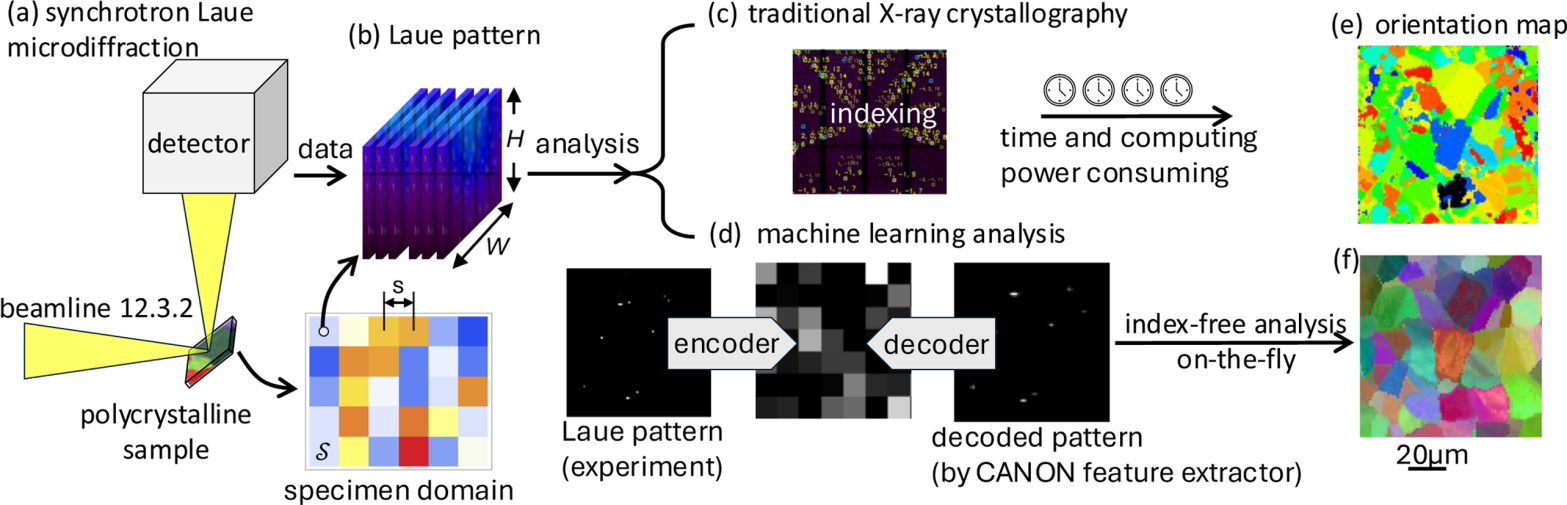
Schematics of the synchrotron X-ray Laue microdiffraction experiment. (*a*) Experiment in beamline, which generates a sequence of (*b*) Laue patterns corresponding to the 2D specimen domain, 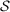, with a scanning step distance, *s*. (*c*)–(*e*) Illustrate the traditional data analysis procedure by X-ray crystallography, while (*d*)–(*f*) elaborates on the ML approach to analyze the Laue microdiffraction data through feature extraction and unsupervised learning.

The standard equipment setup and data analysis of the synchrotron X-ray microdiffraction experiment is illustrated in Fig. 1 ▸. We perform a 2D raster scan on the specimen domain defined as 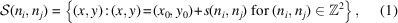
where the vector (*x*_0_, *y*_0_) specifies the starting location on the sample surface associated with the left-upper corner in the specimen domain, the value *s* > 0 denotes the scanning step size of the focused X-ray beam, and the range of integer tuples (*n_i_*, *n_j_*) underlies the total number of Laue patterns collected in each of the microdiffraction experiments. Mathematically, the microdiffraction experiment is a mapping, 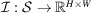, where *H* and *W* are the pixelate dimensions of a Laue pattern image [Fig. 1 ▸(*b*)]. For 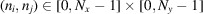, the specimen domain is mapped to *N_x_N_y_* Laue pattern images. For 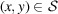, the Laue pattern can be expressed as 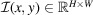.

If the grain size is larger than the X-ray beam size, the scanning step, *s*, can be set to be at least the beam size. Then the Laue patterns in the scanning sequence are sufficient to distinguish the orientation difference across the grain boundaries by traditional X-ray crystallographic analysis [Figs. 1 ▸(*b*) → 1 ▸(*c*)]. Finally. an orientation map for the polycrystal domain is generated [Fig. 1 ▸(*e*)]. Note that the grain boundaries resolved by Laue indexing are not spatially precise as the Laue patterns collected near the grain boundary consist of the *hkl* peaks diffracted by both neighboring grains. By ML-assisted analysis (Song *et al.*, 2019 ▸) [Figs. 1 ▸(*b*) → 1 ▸(*d*)], the grain boundaries in the scanned domain can be resolved more precisely [Fig. 1 ▸(*f*)].

**Figure 2:**
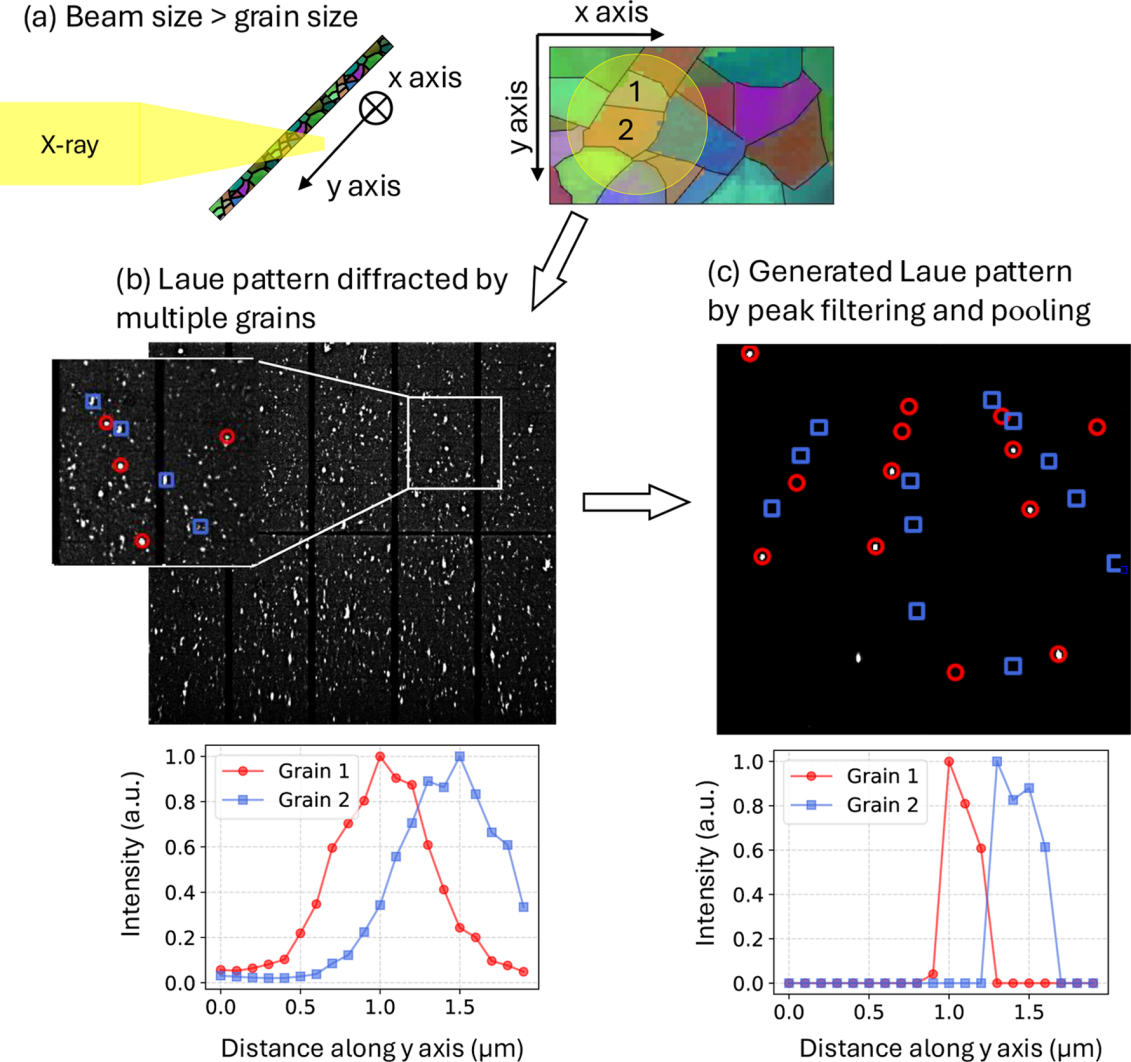
Comparison between the Laue pattern collected from experiment and the Laue pattern generated by a peak-pooling algorithm. (*a*) Illustration of the incident X-rays with a beam size greater than the grain size. (*b*) Original Laue pattern produced by diffraction by multiple grains with different orientations. The intensity profiles of grain 1 and grain 2 are spatially unresolvable. (*c*) Artificial Laue pattern generated by equation (2), mainly showing the reflections from grain 1. The intensity profiles of grain 1 and grain 2 are better spatially resolvable.

If the grain size is smaller than the X-ray beam size but larger than the scanning step size, the diffraction peaks in neighboring Laue patterns will overlap. For example, at the microdiffraction beamline 12.3.2 at the ALS, Lawrence Berkeley National Laboratory, the precise motorized stage can achieve a scanning step of *s* ≥ 100 nm with the X-ray beam focused to about 1 µm. The beam profiles in both the horizontal and vertical directions follow a Lorentzian distribution, whose slow decay ensures significant diffraction even well beyond the focal point. Though the profile shape remains stable, gradual degradation of the Kirkpatrick–Baez mirrors causes the full width at half-maximum (FWHM) to drift, currently measuring approximately 1.2 µm horizontally and 1.0 µm vertically. If the grain size is approximately 500 nm, the neighboring Laue patterns along the scanning sequence may consist of peaks diffracted by the same grain, illustrated in Fig. 2 ▸(*a*). In this case, direct crystallographic analysis may not resolve the spatial boundaries between grains with different orientations. Even if we can index the highly entangled Laue pattern with advanced indexing algorithms (Tamura, 2014 ▸; Purushottam Raj Purohit *et al.*, 2022 ▸; Seret *et al.*, 2022 ▸; Kacprzak *et al.*, 2024 ▸), the intensity profiles shown in Fig. 2 ▸(*b*) reveal that grain 1 (red) and grain 2 (blue) are still unresolvable. Nevertheless, the geometric features and intensity profiles of each Laue peak possess subtle differences. Such subtle differences can be used to differentiate the spatial domain with proper *learning* of the latent features for the Laue pattern.

### 2.2. Physics-informed peak filtering and pooling method

**Figure 3:**
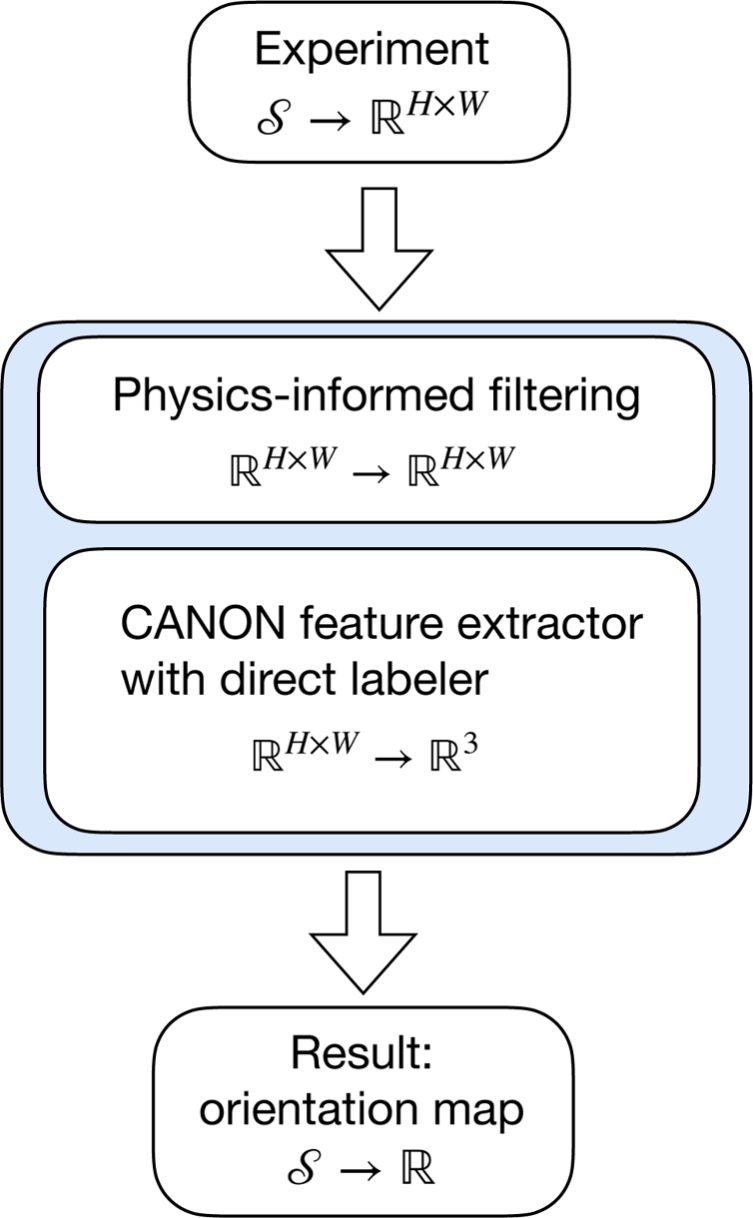
PIML workflow for grain mapping by synchrotron X-ray Laue microdiffraction.

Utilizing the intensity amplitude and profile of each Laue peak instead of indexing the entire Laue pattern, the illuminated portion of each grain exposed by the micrometre-sized X-ray beam can be revealed by a PIML workflow, illustrated in Fig. 3 ▸. For every spatial location, 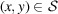, the experimental Laue pattern 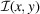 can be mapped to an artificial Laue pattern,
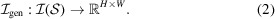
The algorithm to calculate 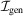 involves two main steps. To enhance the spatial resolution of nanoscale grain mapping, we implemented an adaptive mask computation strategy for filtering Laue diffraction peaks. In the neighborhood of each Laue pattern 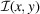 within a radius of (*s*, *s*) for a Laue microdiffraction scan, we identify the peak with the highest intensity, *i*_max_. For all peaks in 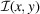, we block the peaks if their intensities are less than 80% of *i*_max_. This approach effectively suppresses weaker, less significant peaks originating from satellite grains while preserving the dominant reflections from the primary grain in the exposure domain. This computed mask helps to filter out the high-intensity peaks from satellite grains within the neighborhood of the measured Laue pattern. When the grain size distribution is heterogeneous so that a large grain lies beneath several smaller ones, the large grain may dominate the reflections in the artificial Laue pattern given in equation (2). After filtering and pooling, the generated Laue pattern always comprises reflections from the grain that occupies the largest volume exposed by the synchrotron beam. Consequently, the algorithm infers a grain morphology bounded by the largest illuminated grains, which may lead to an overestimate of the actual grain sizes in the material.

Following the mask computation, we proceed with peak filtering and pooling, an algorithm that aligns with the nature of X-ray diffraction by crystals. By leveraging the inherent characteristics of diffracted peaks, this method effectively distinguishes the diffraction patterns from neighboring grains. As a result, the complex Laue patterns are significantly clarified, making them much cleaner and more suitable for further analysis. Fig. 2 ▸(*c*) demonstrates the generated artificial Laue pattern after peak filtering and pooling, highlighting that grain 1 (red) predominantly contributes to the primary reflections, in contrast to grain 2 (blue). The intensity profiles of the generated artificial pattern, 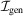, reveal improved resolution of the two grains within the spatial specimen domain along the *y* axis.

We used the Laue pattern autoencoder *CANON* (Song *et al.*, 2019 ▸) to extract latent features from the generated Laue patterns. Subsequently, we applied an indexing-free ML algorithm (Song *et al.*, 2019 ▸) to segment the specimen domain, 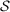, on the basis of the pairwise distances in the reduced-dimensional latent feature space. In particular, the feature vectors are reduced to three dimensions (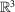) by PCA. Each component of the feature vector is represented by one of the RGB colors that we use to construct the grain map from the topological clustering results, as elaborated in Fig. 3 ▸.

## 3. Results and discussion

We demonstrate our PIML method by characterizing a nanocrystalline Au thin film on a silicon substrate. The film was fabricated by electron beam evaporation on a cleaned single-crystal silicon substrate, resulting in a thin film with a thickness of approximately 1 µm and grain sizes in the nanometre range. We conducted synchrotron X-ray Laue microdiffraction on the sample surface at Beamline 12.3.2 of the ALS, Lawrence Berkeley National Laboratory. The focused X-ray beam had a nominal beam size of 1 µm. A two-dimensional scan using a polychromatic X-ray beam in the energy range 6–22 keV was performed with a step size of 100 nm, systematically capturing Laue diffraction patterns across the specimen domain.

Since the thin layer of Au does not fully diffract all X-rays, the Laue patterns from the specimen domain exhibited very strong silicon reflections. To address this, we conducted a background filtering process to suppress the silicon reflections, ensuring clearer patterns for analysis. This preprocessing step was essential before applying the PIML analysis for nanocrystalline mapping.

### 3.1. Grain mapping of Au nanocrystalline thin film

**Figure 4:**
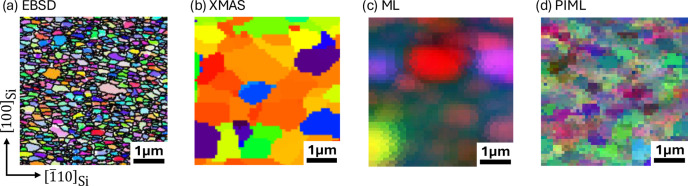
Grain maps generated by different methods. (*a*) Orientation map by EBSD. (*b*) Orientation map by X-ray Laue microdiffraction analyzed by the *XMAS* software. Grain topographic maps generated from the same microdiffraction dataset by the (*c*) indexing-free ML algorithm and (*d*) PIML algorithm.

Electron backscatter diffraction (EBSD) was performed on the same specimen using a JEOL JSM-7800F scanning electron microscope. The scan, conducted with a 25 nm step size over a 20 µm × 20 µm area, utilized a 20 kV high-energy electron beam to differentiate grains via backscattering. The interaction of the electron beam with the crystal structure generated characteristic Kikuchi patterns, which were analyzed using the point group *m*3*m* to create the orientation map shown in Fig. 4 ▸(*a*) with the horizontal direction aligned with the [110] direction of the single-crystal silicon substrate. Post-processing with the *AZtecCrystal* software (https://nano.oxinst.com/azteccrystal) provided precise grain orientations and morphological details, including grain size and orientation distributions, represented by different colors. Au nanograins exhibit anisotropic morphology, elongated along the horizontal direction ([110_Si_]). We performed synchrotron X-ray Laue microdiffraction on the same sample with identical alignment to examine the orientation distribution and morphology by ML methods. While the EBSD-characterized region does not perfectly overlap with the area measured by X-ray microdiffraction, the grain morphologies from the two experiments are comparable due to the randomness in orientation distribution in the Au thin film.

We utilized *XMAS* to index each of the Laue patterns using traditional crystallographic analysis, resulting in the grain mapping shown in Fig. 4 ▸(*b*). However, the Laue indexing process was insufficient in accurately determining grain boundaries among nanosized grains. This limitation arises from the fact that each Laue pattern consists of diffraction peaks from many satellite grains, and the micrometre-sized X-ray beam cannot spatially resolve the grain boundaries. Consequently, *XMAS* struggled to distinguish nanosized grains, leading to incomplete or blurred grain boundary definitions.

For comparison, Fig. 4 ▸(*c*) shows the grain mapping produced by the indexing-free ML algorithm without physics-informed filtering and pooling processes (Song *et al.*, 2019 ▸). While the ML approach offered an alternative method, it similarly failed to identify nanosized grains, highlighting the complexity of accurate grain boundary detection at this scale.

In contrast, the grain mapping generated by the PIML method, shown in Fig. 4 ▸(*d*), successfully reveals fine grains at scales of several hundred nanometres. This result aligns closely with the morphology obtained from EBSD in Fig. 4 ▸(*a*), demonstrating the effectiveness of the PIML approach in resolving nanoscale features. Additionally, the nanocrystalline Au grains exhibit noticeable anisotropy, with most grains elongated along the horizontal direction. This anisotropic feature is evident in both Figs. 4 ▸(*a*) (EBSD) and 4 ▸(*d*) (PIML), further confirming the consistency between these methods.

### 3.2. Grain morphology analysis

**Figure 5:**
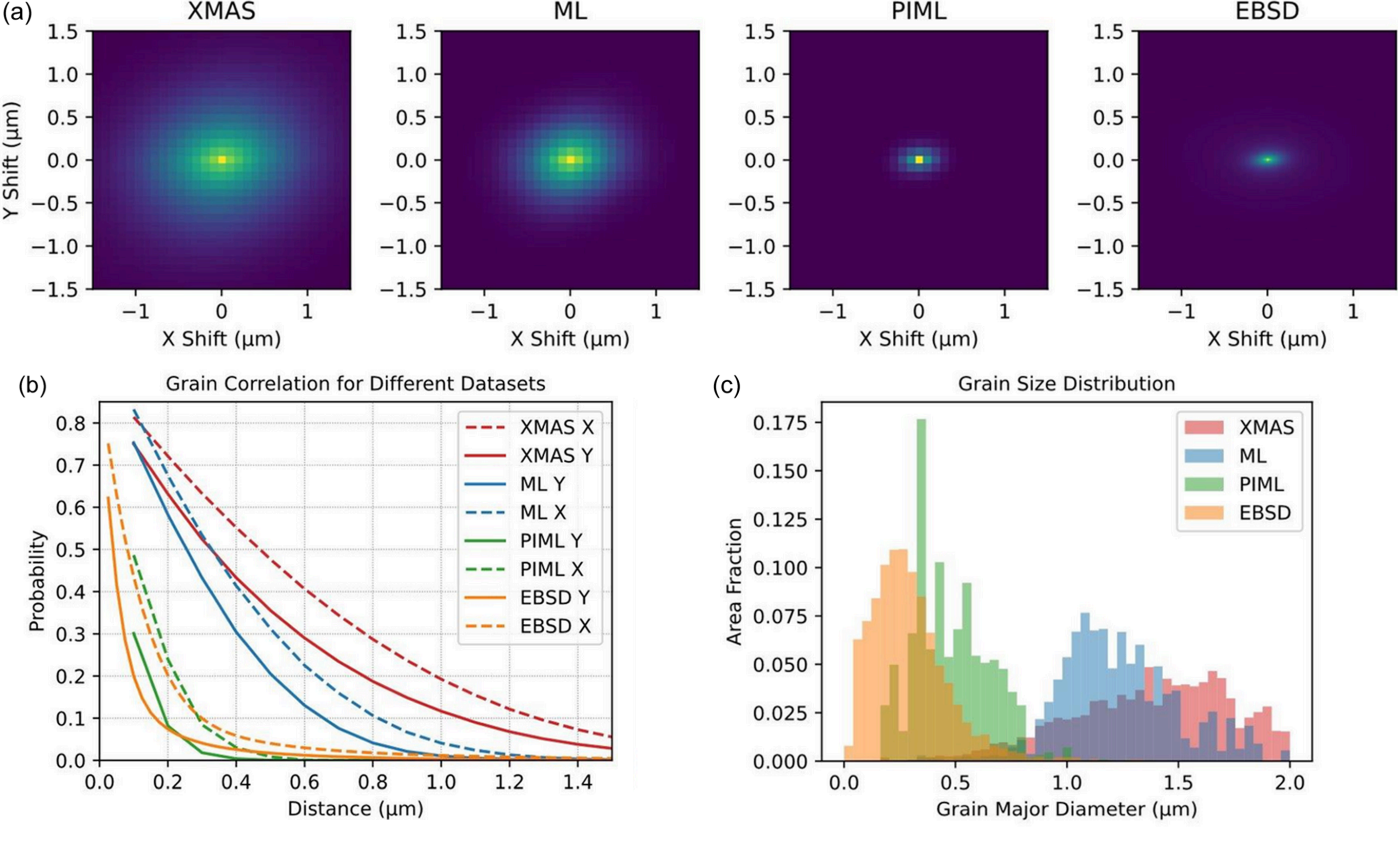
Quantitative assessments of grain segmentation by different analysis methods. (*a*) Two-point correlation of the grain maps given by different methods. (*b*) Grain size distribution along the *x* and *y* directions corresponding to (*c*) expectations and variances of grain morphologies.

A quantitative comparison between the grain maps given by different methods in Fig. 4 ▸ was performed using a two-point correlation analysis, as illustrated in Fig. 5 ▸. The two-point correlation function [Fig. 5 ▸(*a*)] is a statistical measure that calculates the probability of any two points within the specimen domain belonging to the same grain. To assess grain size using this function, we calculate the probability for each pair of points and plot it as a function of the distance between the points [Fig. 5 ▸(*b*)]. The rate at which this function decays with distance provides information about the grain size: a diffusive decay indicates larger grains, while a sharp decay suggests smaller grains. By fitting the correlation function, we can extract parameters that describe the anisotropy of grain morphology [Fig. 5 ▸(*a*)] and grain size distribution including the mean grain size and variance [Fig. 5 ▸(*c*)].

The grain morphology obtained by *XMAS* and ML without physics-informed filtering exhibits a Gaussian shape with an FWHM of approximately 1 µm, close to the size of the X-ray beam, indicating that the spatial resolution of these methods is limited by the beam size. In contrast, the grain morphology given by PIML is similar to that given by the EBSD scan, with an FWHM of approximately 0.3 µm. This suggests a better spatial resolution beyond the beam size limit.

The grain maps characterized by EBSD and analyzed by PIML algorithms both exhibit grain anisotropy, with a higher correlation observed in the *x* direction compared with the *y* direction. This indicates that the grains are predominantly elongated horizontally (*i.e.* along the *x* direction). The PIML method captures this grain anisotropy more effectively than other analysis methods. The grain size distribution, expectation and variance of the grain map given by PIML match the EBSD data very well. Specifically, both methods show a Poisson distribution, with a mean grain size of approximately 300 nm for EBSD and 480 nm for the PIML method.

While the PIML algorithm improves the effective spatial resolution, it still slightly overestimates the mean grain size compared with that measured by EBSD. One possible reason for this discrepancy is the residual influence of the beam size and step size. Additionally, the Laue mask used in equation (2) for physics-informed filtering plays a crucial role in determining the grain boundaries. Since Laue scanning takes several hours to complete, experimental noise and variations in peak intensity measurements could also contribute to this discrepancy.

## 4. Conclusions and outlook

The PIML method enhances the spatial resolution of Laue microdiffraction characterization for nanocrystalline polycrystals. While powder diffraction with a large monochromatic beam can provide accurate average grain size and texture information, Laue microdiffraction offers additional insights such as grain boundary morphology and orientation distribution. It also provides information on crystallographic defects within a grain like twinning, slip lines and stacking faults. Moreover, Laue microdiffraction has advantages over EBSD, including strain sensitivity, the absence of the need for additional sample preparation and vacuum compatibility. This improvement significantly impacts materials characterization, as accurate knowledge of grain size distributions and orientations is crucial for understanding material properties. Future work could focus on optimizing the physics-informed hyperparameters and exploring the algorithm’s applicability to various materials under different experimental conditions and crystal symmetries.

## Data Availability

The data supporting the results are all reported in the article.

## Footnotes

1 The *CANON* package includes the Laue pattern autoencoder, pre-imaging processing, peak filtering and pooling, and principle component analysis (PCA)-based segmentation, and is implemented in Python. To date, the code has not been released publicly since it was developed specifically for data collected at ALS beamline 12.3.2. In future we plan to build a general purpose portal to make *CANON* widely available.
